# The Effect of Fine-Ground Glass on the Hydration Process and Properties of Alumina-Cement-Based Composites

**DOI:** 10.3390/ma14164633

**Published:** 2021-08-17

**Authors:** Galyna Kotsay, Irmina Masztakowska

**Affiliations:** Faculty of Civil Engineering, Mechanics and Petrochemistry, Warsaw University of Technology, Łukasiewicza St. 17, 09-400 Płock, Poland; irmina.masztakowska.stud@pw.edu.pl

**Keywords:** high-alumina, calcium aluminate cement, fine-ground glass, paste, mortar

## Abstract

This paper discusses studies regarding the impact of fine-ground glass additives on the hydration and properties of alumina cement pastes and mortars. Fine-ground glass was added to pastes and mortars instead of high-alumina cement and calcium aluminate cement in quantities of 5% and 10%. The findings are inconclusive as to the impact of glass on the properties of tested alumina cement types. The effect produced via the addition of glass instead of cement depends on the type of alumina cement used. Adding fine-ground glass to high-alumina cement enhances the paste’s density while improving paste and mortar strength. Using the same additive for calcium aluminate cement reduces its density and strength. The addition of glass to high-alumina cement adversely affects its strength at higher temperatures.

## 1. Introduction

Alumina cement is a type of cement characterized by very high early-stage hydration heat and demonstrates a rapid gain of strength. The increased hydration heat characteristic of alumina cement (also known as self-heating) allows for the pouring of concrete at low temperatures. In contrast to Portland cement, alumina cement exhibits superior durability in aggressive environments due to the absence of portlandite and ettringite from the hydration products [1,2,3,4,5]. However, a disadvantage of alumina cement is the conversion process, in which unstable low-density calcium aluminate hydrates turn into durable, high-density hydrates. Such an increase in density may reduce the strength of the products. These density changes are considered inevitable and irreversible but necessary to achieve alumina cement’s desired properties [6,7]. The following conditions must be met to avoid the adverse effects of incorrect conversion: temperature must be kept above 38 °C [8,9,10,11], an adequate water/cement ratio must be provided [2,9], and cement paste alkalinity needs to be maintained at the appropriate level. In addition, conversion needs to occur as the cement paste is hardening, not later [6,12,13,14]

In many articles, researchers have considered the possibility of reducing the conversion of cement hydration products by using mineral additives such as blast furnace slag, fly ash, silica fume, metakaolin and zeolite [11,12,15,16,17,18,19,20,21,22,23,24,25]. Replacing part of alumina cement with these mineral additives reduces the heat of hydration and causes a reduction in strength. According to the authors, mineral additives react with unstable products of cement hydration CaO·Al_2_O_3_·10H_2_O (CAH_10_) and 2CaO·Al_2_O_3_·8H_2_O (C_2_AH_8_) (In this paper abbreviations are used according to Standard cement chemistry: C = CaO, A = Al_2_O_3_, H = H_2_O, S = SiO_2_, N = Na_2_O) to form a stable product, stratlingite 2CaO·Al_2_O_3_·SiO_2_ 8H_2_O (C_2_ASH_8_), which does not change its density over time, therefore preventing a possible decrease in mechanical strength.

To inhibit conversion, the action of mineral additives alone is insufficient. Thus, some authors have suggested using mineral additives along with alkaline compounds [20,26,27,28,29,30]. The high pH generated by alkaline solutions accelerates the conversion process even at ambient temperatures. Therefore, as reported in [26], the formation of stable stratinglite (C_2_ASH_8_) and hydrogarnet (C_3_AH_6_) phases resulted from the alkaline activation of the additives. However, these products show lower strength values in the early stages of cement hardening. The alkaline activation of aluminum silicate additives may also result in the formation of the sodium-aluminum silicate hydrate product (NASH gel), also known as an inorganic alkaline polymer [31,32,33,34]. According to [35], research exists on the alkaline activation of alumina cement itself. However, alkali content in alumina cement should, as a standard, be below 0.5% because of the possibility of forming easily soluble alkali aluminates. However, the acceptable alkali limit content according to [36] depends on the form in which the alkali appear in the clinker.

Many works have proposed fine-ground glass instead of Portland cement [37,38,39,40,41,42,43,44,45,46,47,48] or geopolymer cement [49,50,51,52,53,54,55,56]. However, in the literature, there is a lack of knowledge concerning the influence of fine-ground glass on the properties of alumina cement. In connection with the above, the purpose of this work was to investigate the effects of fine-ground glass additive on the hydration and properties of calcium-alumina and high-alumina types of cement. The materials containing different amounts of waste glass were characterized via the apparent density, X-ray diffraction, and the compression testing of pastes and mortars stored at various temperatures.

## 2. Materials and Methods

**Figure 1:**
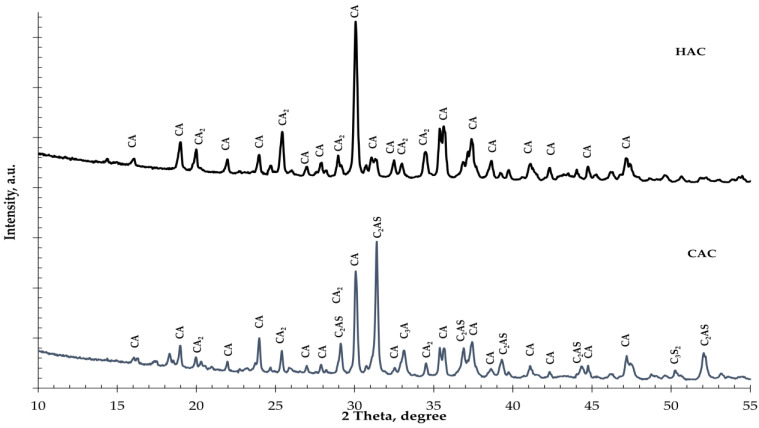
The X-ray diffraction results of cement types: High-alumina cement (HAC); calcium aluminate cement (CAC). (CA—CaO·Al_2_O_3_; CA_2_—CaO·2Al_2_O_3_; C_2_AS—2CaO·Al_2_O_3_·SiO_2_; C_3_A—3CaO·Al_2_O_3_; C_3_S_2_—3CaO·2SiO_2_).

Two types of alumina cement were used in the study: high-alumina cement (HAC) produced by Górka Cement (Trzebinia, Poland) [57] and calcium aluminate cement (CAC) produced by the Rongsheng (ZhengZhou, China) [58]. The mineralogical composition of the tested types of cement is shown in Figure 1. Table 1 lists the identified primary and associated phases. The chemical composition and properties of alumina types of cement and fine-ground glass are presented in Table 2. Chemical analysis of alumina cement was conducted following the standard [59], and glass was carried out according to [60]. Fine-ground glass with a grain size of ≤0.063 mm was used as an additive instead of cement in 5% and 10% amounts.

The real density and the Blaine specific surface test of materials were performed according to the methods described in the standards [61,62].

Apparent density tests were carried out according to [61]. The apparent density and the compressive strength of the cement pastes were tested on cubic specimens. Cementitious pastes were prepared using at fixed water-to-cement ratio as w/c = 0.3. In order to eliminate defects, specimens of paste were formed in steel molds with precise dimensions cubies 20 mm × 20 mm × 20 mm. This made it possible to compact them precisely after the cubies were filled with paste. The curing of the paste specimens was carried out in enclosed molds for the first 24 h, followed by immersion of the cubic specimens underwater. These were cured at 20 °C and 40 °C in the thermostated chamber until testing. The specimens were stored at 40 °C to prevent uncontrolled conversion effects. Every property of paste analyzed was evaluated on a series of six specimens. The tests were performed at 2, 5, and 28 days after forming.

The water/cement ratio (w/c) was 0.4 for the mortars prescribed by [6]. According to [63], the mortar strength test was carried out on specimens sized 40 mm × 40 mm × 160 mm. The curing of the mortar specimens was carried out similarly to the paste specimens. Tests for flexural strength and compressive strength were carried out after 2 and 5 days.

Calorimeter Calmetrix I-Cal 2000 HPC was applied to determine the effect of glass on the hydration of aluminate cement. The rate of heat evolution was recorded for the cement pastes with a water-to-binder ratio equal 0.5. Binder mixtures were made from cement and glass as partial cement substitution. The rate of heat evolution was measured at the isothermal condition (20 °C) and recorded for 48 h.

Qualitative X-ray diffraction analyses were carried out on HAC and CAC cement pastes after 365 days of hydration. The XRD was conducted using a Cubix Pro PANalytical diffractometer with Cu Ka radiation (λ = 1.54 Å). The samples were scanned between 10° and 65° (2 Theta), with scan step of 0.021° (2 Theta) and time step 30 s. Qualitative X-ray diffraction analyses of crystalline phases were sourced from the standard databases [64].

## 3. Results and Discussion

**Figure 2:**
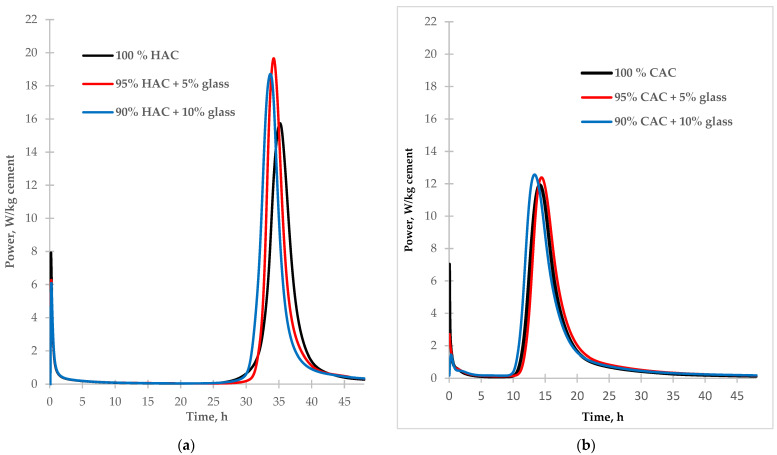
The calorimetric curves of hydration of the tested types of cement HAC (**a**) and CAC (**b**) (with and without glass).

Two types of alumina cement with different mineral compositions were used for the tests. In high-alumina cement (HAC), the basic mineral components are monocalcium aluminate (CA) and dicalcium aluminate (CA_2_), while in calcium aluminate cement (CAC), these are gehlenite (C_2_AS) and monocalcium aluminate (CA). During the hydration of monocalcium and dicalcium aluminate, unstable hexagonal phases are formed which undergo a conversion process to create the stable phases of calcium hydroaluminate and aluminum hydroxide [1,2]. During the hydration of gehlenite (C_2_AS), only the stable stratinglite (C_2_ASH_8_) phase was formed. High-alumina cement is more susceptible to conversion than CAC due to its higher mineral monocalcium aluminate and dicalcium aluminate contents. The calorimetric study results of the investigation into the effect of fine glass on the hydration of two different types of aluminous cement are presented in Figure 2.

According to the data in Table 3, the addition of glass as a partial replacement for alumina cement increases the amount of hydration heat released from cement pastes after 48 h. Compared to control samples, replacing 10% of the cement with glass increased hydration heat after 48 h by 4% for high-alumina cement and 8% for calcium aluminate cement. In each alumina cement paste sample, the introduction of glass reduces the heat release rate in the pre-induction stage of hydration due to decreased cement content in the mixture. However, the addition of glass increases the rate of cement hydration in the post-induction stage. The highest rate of heat flow was noted for HAC paste (Figure 2a). The heat flow rate rose by 25% and 20% with 5% and 10% glass, respectively. The heat flow rate increase was probably related to the reaction of the glass with the cement hydration products. For the CAC paste (Figure 2b), with the addition of glass, the quantity of heat flow in the post-induction stage of hydration increased only slightly compared to the control sample.

It is known that the transformation of two unstable alumina cement hydration products (CAH_10_ and C_2_AH_8_) into the stable phase (C_3_AH_6_) is considered inevitable and irreversible. The phase C_3_AH_6_ has a higher density than the CAH_10_ and C_2_AH_8_ phases by 42% and 30%, respectively (Table 4). The hydration products’ conversion rate is impacted by the temperature, water-to-biller ratio, and the binder’s alkali content. As it is known, waste glass contains alkali in the amount of 13%. Therefore, the introduction of waste fine-ground glass instead of alumina cement may affect the conversion process of unstable cement hydration products.

**Figure 3:**
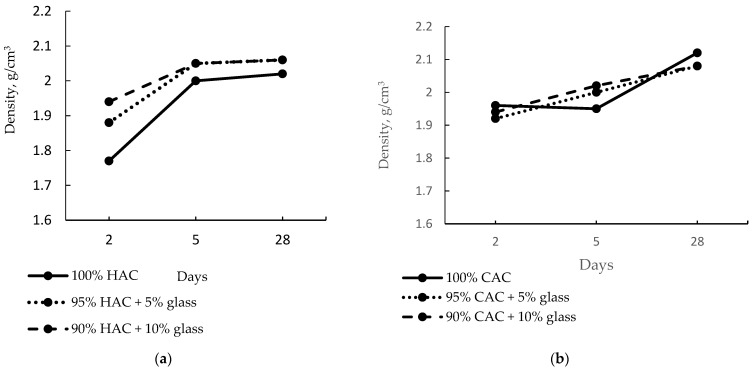
The density of pastes of HAC (**a**) and CAC (**b**) stored at 20 °C.

Figure 3 shows the effect of fine-ground glass on the density of the alumina pastes. The introduction of 5% and 10% of glass increases the high-alumina paste density after just two days to 6% and 10%, respectively.

After five days of hardening, the density increased to 13% for the control paste sample and only 6% for the paste with 10% glass content, proving that the fine-ground glass accelerates the conversion process of HAC cement hydration products. Therefore, it shows a minor increase in density. However, after 28 days of hardening, no change was recorded in the pastes’ density containing 5% and 10% glass content. In the CAC case, the density of the control paste was higher than that of the HAC paste because the CAC hydrates faster than the HAC due to the lower CA_2_ content, which hydrates very slowly (Figure 2). Therefore, the introduction of fine-ground glass instead of the CAC did not significantly alter the paste’s density after two days compared to the control sample (Figure 3b). However, after 28 days of hardening the pastes prepared from CAC, a drop in density was observed for pastes with 5% and 10% glass content compared to the control sample.

**Figure 4:**
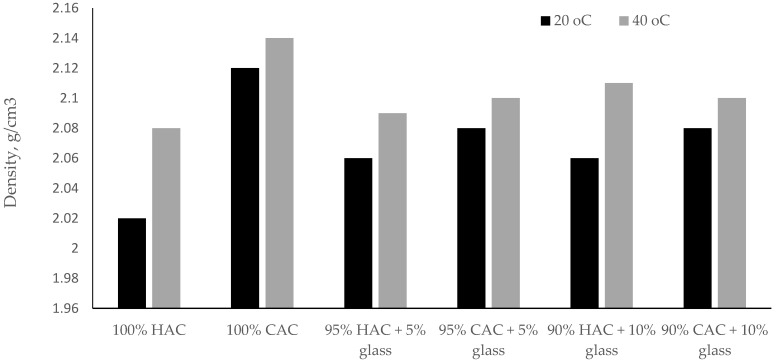
The density of pastes after 28 days under temperatures of 20 °C and 40 °C.

Raising the storage temperature of the specimens pastes to 40 °C increases the density of high-alumina cement pastes. On the other hand, for calcium aluminate cement pastes, the density increase was insignificant compared to high-alumina cement (Figure 4). These results indicate that the tested binder CAC is less susceptible to the conversion process compared to binder HAC.

**Figure 5:**
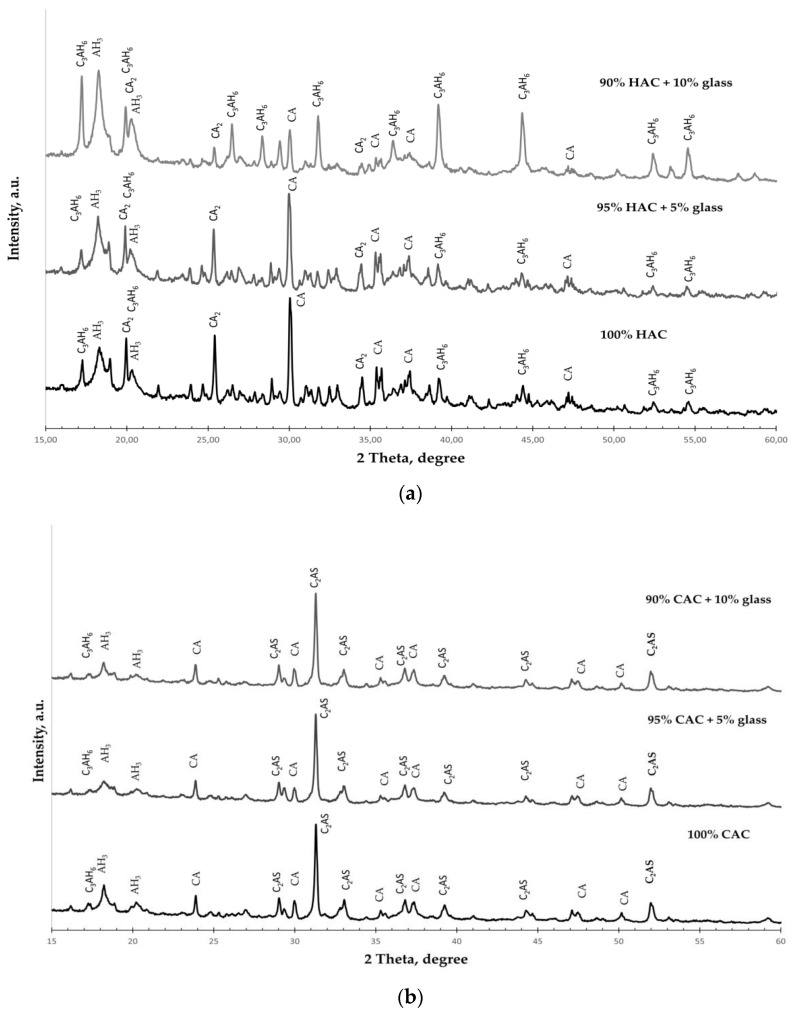
The XRD spectra for the pastes hydrated for 365 days. (**a**) HAC-glass systems; (**b**) CAC-glass systems.

XRD was performed to analyze the phase transformations of the studied types of cement hydration products. Figure 5 shows the XRD spectra of cement pastes with different glass contents after 365 days of hardening.

The phases are identified as follows: hydrogarnet (C_3_AH_6_); gibbsite (AH_3_); monocalcium aluminate (CA); dicalcium aluminate (CA_2_), gehlenite (C_2_AS).

X-ray diffraction spectra of the HAC-glass pastes (Figure 5a) show that the dominant phases were hydrogarnet (C_3_AH_6_) and gibbsite (AH_3_). Simultaneously, for plain HAC, there are unhydrated phases of CA and CA_2_, even after 365 days of hardening. These phases are fewer, but there are more C_3_AH_6_ and AH_3_ phases in HAC systems with ground glass. The amount of C_3_AH_6_ increases with the increasing amount of glass, confirming the significant increase in the C_3_AH_6_ peaks in the XRD pattern. These results show that additive glass accelerates the conversion process in HAC systems.

However, in the XRD spectra of the CAC-glass pastes, unhydrated phases of gehlenite (C_2_AS) and dicalcium aluminate (CA_2_) are observed even after 365 days of hardening (Figure 5b). The hydrated phases observed are hydrogarnet (C_3_AH_6_) and gibbsite (AH_3_), and there are probably other hydration products of the CAC binder that are poorly crystallized. It is thus challenging to identify them via XRD analysis. The size of the AH_3_ phase decreases slightly with the increase in the amount of glass in the system, which is confirmed by the decrease in the intensity of the AH_3_ peaks in the XRD pattern.

In conclusion, glass addition significantly influences the acceleration of the conversion process for the HAC systems. However, there are no significant changes for the CAC system, as evidenced by the tests of cement paste density.

**Figure 6:**
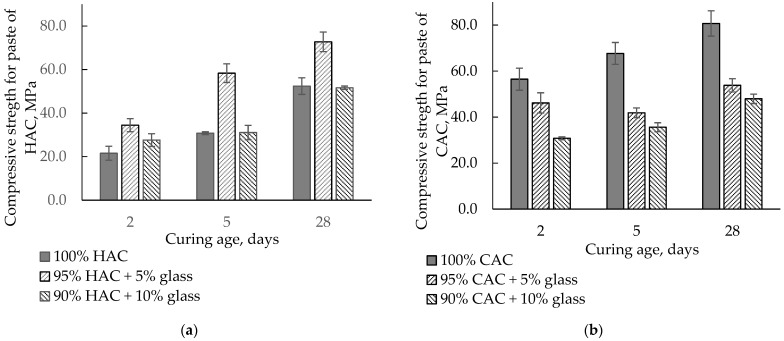
The compressive strength for the paste of HAC and CAC with glass stored at 20 °C. (**a**) HAC-glass systems; (**b**) CAC-glass systems.

Figure 6 presents the influence of HAC and CAC replacement by glass on the compressive strength of pastes after 2, 5, and 28 days of hardening. Replacing HAC with 5% glass increases the compressive strength compared to the control sample. The highest increase was observed after 5 days of hardening, while after 28 days, when a stable strength had been obtained, it was higher by approximately 40%. In addition, 10% glass caused an increase in strength after two days, while after 5 and 28 days, the compressive strength was comparable with the control sample. The increased strength of HAC pastes was associated with an acceleration of the hydration process of the HAC binder with the addition of glass.

On the other hand, the replacement of CAC with glass significantly decreased paste strength because glass slightly affects the CAC’s hydration and reduces the amount of binder in the paste. With 10% glass instead of cement, the strength falls after 5 and 28 days by 45−50% from the control sample values.

**Figure 7:**
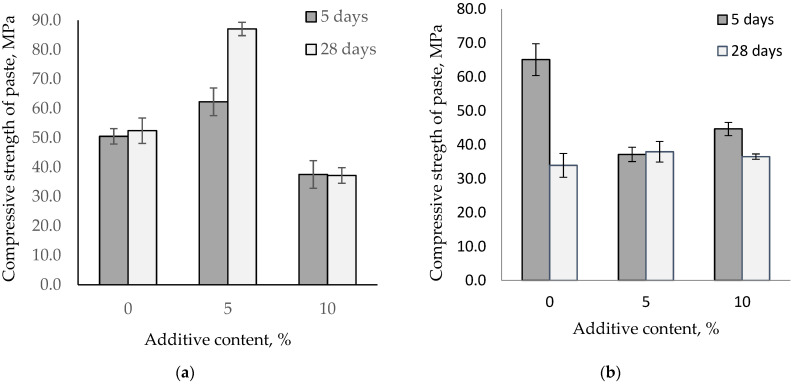
Compressive strength for the paste of HAC and CAC with glass stored at 40 °C. (**a**) HAC replacement by glass; (**b**) CAC replacement by glass.

It is known that increasing the temperature accelerates the conversion of metastable hydrates CAH_10_ and C_2_AH_8_ into the stable hydrate C_3_AH_6_. Depending on the alumina cement mineral composition, the conversion process rate varies, thereby affecting the paste’s properties. Figure 7 shows the influence of fine-ground glass on the change in the compressive strength of alumina pastes stored at 40 °C.

Increasing the storage temperature to 40 °C leads to changes in the strength of alumina pastes. After the first five days of hardening, an increase in the temperature strengthens high-alumina cement and calcium aluminate cement pastes. However, after 28 days, there is a difference across the alumina pastes. The strength of the high-alumina paste is comparable to the strength after five days, while for the control calcium aluminate paste, the strength had nearly halved. The high-alumina cement with 5% glass content increases cement strength by 23% and 66% after storage at a temperature of 40 °C. However, pastes with 10% glass content experience a reduction in strength compared to the control samples. For calcium aluminate cement, the use of glass stabilizes the strength under the influence of higher temperatures.

**Figure 8:**
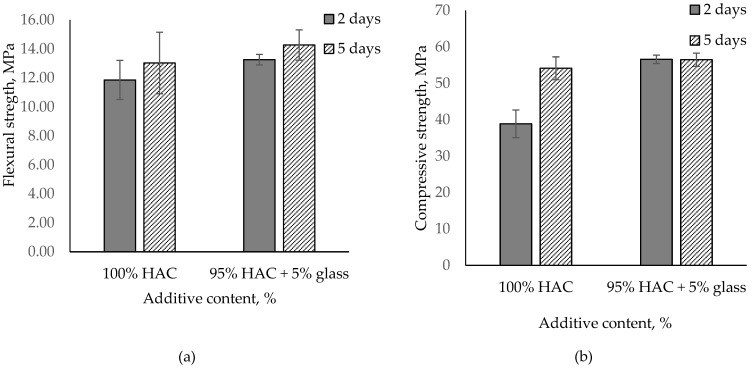
Flexural and compressive strength for the mortar paste of HAC with glass stored after 2 and 5 days. (**a**) Flexural stregth; (**b**) Compressive stregth.

Based on the analysis of the results of the tested cement paste, the effect of glass on alumina cement properties depends on the types of cement used, with the impact on paste and mortar properties being proportional to mono- and dicalcium aluminate content in the cement. The usage of glass instead of HAC in paste accelerates the hydration of cement, conversion process and increases compressive strength. In contrast, the use of glass instead of CAC (containing gehlenite) in paste did not significantly accelerate the hydration of cement and decreased compressive strength. Increasing the glass content lowers the compressive strength of the paste; accordingly, 5% glass content was added to the mortar, replacing HAC. Figure 8 presents the influence of HAC replacement with glass on the flexural and compressive strength of pastes after 2 and 5 days of hardening.

Just as for pastes, high-alumina mortars with 5% glass content were found to have grown in compressive strength by 46% after two days. The results, however, levelled off to control values after five days. Flexural strength also increased from 10% to 12% after 2 and 5 days, respectively (Figure 8).

## 4. Conclusions

Based on the analysis of the obtained research results, the following conclusions can be drawn:

The effect of glass on the properties of alumina cement types depends on the cement used, with the impact on paste and mortar properties being proportional to the mono- and dicalcium aluminate content in the cement.

The calorimetric test results showed an increase in the rate of heat emission for alumina cement pastes with the addition of fine-ground glass in the post-induction period. The highest heat emission rate was recorded for the high-alumina cement paste, with the heat emission rate rising to 25% for 5%-glass content and 20% for 10%-glass content.

Compared to the control pastes, the use of glass instead of high-alumina cement causes an increase in the density of the pastes, while replacing the calcium aluminate cement with glass causes a decrease in the density of the pastes.

Adding fine-ground glass to a content of 5% to pastes containing high-alumina cement increases the compressive strength significantly at 20 °C. After 28 days, the increase in the strength of the pastes equals approximately 40%. Increasing the maturation temperature to 40 °C additionally increases the strength of the pastes to 66%. However, the strength of the pastes with the quantity of glass equal to 10% was comparable to that of the control samples. The compressive strength of mortars with 5% glass increased by 46% after two days and 4% after five days.

For pastes with calcium aluminate cement, the addition of glass is not favorable. Consequently, the addition of glass to a content of 10% instead of cement had the effect of reducing strength after 5 and 28 days by up to 45–50% compared to the control paste samples.

Using fine-ground glass additives instead of high-alumina cement will reduce cement costs and improve its properties, thus enabling broader applications in building construction.

## Figures and Tables

**Table 1:** The mineralogical composition of the tested types of cement.

Cement	Minerals	
	Basic Phases	Associated Phases
HAC	CA, CA_2_	-
CAC	C_2_AS, CA, CA_2_	C_3_S_2_, C_3_A_-_

**Table 2:** The chemical composition and basic properties of cement types and container glasses.

Materials	Oxides (wt %)						Density, g/cm^3^	Specific Surface, m^2^/g
	Al_2_O_3_	CaO	SiO_2_	Fe_2_O_3_	Na_2_O_eq_	SO_3_		
HAC	69.1	29.6	<0.5	<0.3	<0.5		2.96	0.326
CAC	54.5	34.0	8.3	2.30	0.3	0.6	2.95	0.320
Container glasses	1.8	12.0	71.8	0.5	13.1	0.3	2.50	0.341

**Table 3:** The calorimetric characteristics of hydration of the tested types of cement (with and without glass).

Parameter	HAC			CAC		
	No Additive	5% Glass	10% Glass	No Additive	5% Glass	10% Glass
The heat of hardening after 48 h, kJ/kg	262	263	272	251	265	272
The maximum rate of heat evolution in the pre-induction stage of hydration, W/kg	7.93	6.27	6.09	7.03	2.71	1.42
The maximum rate of heat evolution in the post-induction stage of hydration, W/kg	15.74	19.66	18.72	11.93	12.39	12.56

**Table 4:** Density of the main hydration products of aluminous cement [1,2,64].

Phases		Density (g/cm^3^)
CAH_10_	calcium aluminate decahydrate	1.78
C_2_AH_8_	dicalcium aluminate octahydrate	1.95
C_3_AH_6_	hydrogarnet	2.53
AH_3_	gibbsite	2.42
C_2_ASH_8_	stratlingite	2.02

## Data Availability

Data available in a publicly accessible repository.
